# Supplementary material for: Are carbon emissions trading and green financial instruments synergistic? -Comprehensive quantitative research based on content analysis
Source: PLoS One. 2024 Mar 7;19(3):e0298601. doi: 10.1371/journal.pone.0298601 (PMC10919601; doi:10.1371/journal.pone.0298601)
Supplement: S1 Data — (ZIP) [file pone.0298601.s001.zip › Data/地级市Z_F工作报告.zip!/╣≤╓▌/╣≤╤⌠╩╨/└┤╫╘╣1⁄2╓┌║┼í╛┬φ┐╦╩2╛▌═°í┐_2015.docx]

各位代表：

　　现在，我代表市人民政府向大会作工作报告，请予审议，并请市政协委员和其他列席人员提出意见。

　　一、2014年工作回顾

　　过去的一年，在省委、省政府和市委的坚强领导下，在市人大、市政协的监督支持下，我们认真贯彻落实习近平总书记系列重要讲话精神和中央、省委、市委各项决策部署，坚守发展和生态两条底线，坚持主基调主战略和开放带动、创新驱动，积极抢抓新机遇，主动适应新常态，埋头实干，攻坚克难，打造贵阳发展升级版实现良好开局。

　　一年来，我们保持了一个稳步增长的发展速度。全年实现地区生产总值2497.27亿元、增长13.9%，增速继续位居全国省会城市前列。经济总量占全省27%，提高1个百分点，在全省增比进位中位列第一。2000万元口径规模以上工业增加值636.06亿元、增长12.2%。全社会固定资产投资3489.41亿元、增长15.1%。社会消费品零售总额888.58亿元、增长13.1%。外贸进出口总额78.42亿美元、增长24.1%。公共财政预算收入331.59亿元、增长19.6%，公共财政预算支出448.65亿元、增长14%。金融机构存款余额6992.2亿元、贷款余额6560.51亿元，分别增长18.3%和21.3%。城镇常住居民人均可支配收入24961元、农村常住居民人均可支配收入10826元，分别增长9.4%和12.7%。

　　一年来，我们办成了一批标志性的大事。中关村贵阳科技园从无到有、精彩亮相，贵阳综合保税区封关运行，贵州双龙临空经济区启动建设，生态文明贵阳国际论坛写入国家“一带一路”总体规划，打造了全省对外开放合作的制高点。贵广高铁建成通车，贵阳迈入“高铁时代”。创建国家环境保护模范城市顺利通过考核验收。贵阳·贵安国家级大数据产业发展集聚区获批创建。与富士康开展全产业链战略合作。吉利甲醇汽车项目落户贵阳，填补了我市轿车整车制造的空白。首批列入全国移动金融5个试点城市之一，率先发行符合央行标准的移动金融SIM卡。创新城市基层管理“贵阳经验”入选“中国社会治理创新范例50佳”。这些贵阳“好声音”，必将激励着我们在“双赢之路”上阔步前行！

　　一年来，我们深化了一批重点领域和关键环节的改革。扩大“营改增”试点，全面实施工商登记制度改革，新增市场主体5.4万户、增长31%，注册资本1629亿元、增长136%，民营经济占比达51%。加强政府投资年度管理，推进平台公司实体化转型发展。转变产业扶持资金投入方式，市级产业投资基金达9支、规模17亿元。创新城市开发模式，变“生地出让”为“净地出让”，实现“六统一”。南明河水环境综合整治二期成为财政部8个新增PPP示范项目之一。全面完成政府机构改革，大力推进简政放权，取消行政审批事项10项，下放和委托下放41项。在全省率先开展土地承包经营权、农村集体建设用地和宅基地确权登记颁证工作，农房规划建设审批权限依法委托到乡镇。

　　一年来，我们培育了一批具有战略意义的新兴产业。大数据产业风生水起，新注册大数据及关联企业227家，食品安全云、医疗健康云等在全省率先发展，云上企业超过2900家，上线产品达1.4万个；数据中心服务器突破2万台，呼叫中心坐席达2.2万个；全域公共免费WiFi城市项目有序推进，获批创建“宽带中国”示范城市，以大数据为引领的电子信息产业规模达660亿元，被评为“最适合投资数据中心的城市”。医药健康产业不断壮大，集中开工项目36个、占全省的54%。20家医药企业进入全省“巨人计划”培育方阵，30个重点医药品种销售额突破亿元。现代金融业创新发展，贵阳国际金融中心即将实现入驻，贵阳互联网金融产业园形成“前店后厂”发展模式，113家金融机构在筑设立分支机构，全市科技银行达5家，村镇银行实现全覆盖，11家企业在“新三板”挂牌。金融业增加值238.25亿元、增长18.2%。

　　一年来，我们提升了一批具有竞争优势的特色产业。清镇煤电铝、息烽煤电磷等项目有序推进，贵铝电解铝全面复产，贵钢新特材料短流程、黔轮胎异地技改项目顺利投产，福田（云马）新能源客车成功下线，奇瑞轻型客车转产贵阳，装备制造业增加值80.31亿元、增长21.8%。成功举办全省第九届旅游产业发展大会。旅游接待总人次7240.1万、旅游总收入874.39亿元，分别增长20.2%、20%。西南国际商贸城开业运营，中心城区批发业态转移取得阶段性成效，“马上到”国际物流园公路港等项目建成投用，获批全国电子商务示范城市、电子商务与物流快递协同发展试点城市，现代服务业增加值占服务业比重48.7%。20个现代农业高效示范园区加快建设，发展农业龙头企业144家、农产品加工企业40家，新增农民专业合作社113家、家庭农场102个，农产品加工转化率达55%。

　　一年来，我们构建了一个开放合作的新格局。京筑创新驱动、区域合作不断深化，与北京西城区、海淀区、朝阳区结为友好市区，与中关村管委会、北京市科委、投促局、经信委等单位成功举办系列合作活动，继续选派70名干部赴京挂职。积极参与贵广高铁经济带和泛珠三角经济区建设。和广西北海合作共建“北海贵阳港”。深化与港澳台经贸交流合作。与印度班加罗尔市达成结好共识，与爱尔兰米思郡结为友好城市。成功举办生态文明贵阳国际论坛及酒博会、书博会、国际广告节和大数据招商等重大活动。引进阿里巴巴、惠普、中兴、京东、万达、西门子、亿赞普等国际国内知名企业，省外到位资金2260.95亿元，实际利用外资7.62亿美元，分别增长35.7%、20.9%。

　　一年来，我们打造了一批具有高端水平的创新平台。成功组建中科院软件所贵阳分部、贵阳高校科研院所科技创新联盟，成立北京贵阳大数据研究院、贵州大学贵阳创新驱动发展战略研究院，设立北京跨国技术转移协作网络贵阳分中心，建成北京·贵阳大数据应用展示中心。整体提升贵阳国际人才城，17家机构入驻开展服务。引进高层次人才694名，新建“千人计划”专家工作站1个、博士后科研工作站2个。全市国家级研发机构增至16个、省级217个。新增高新技术企业40家、增长23.1%，高新技术工业占规模以上工业增加值比重38.7%。科技进步贡献率达56.4%。

　　一年来，我们实施了一批打基础利长远的重大工程。市域快铁久长至永温货运专线开通运营、贵阳至开阳客运专线全面建成，厦蓉高速公路清镇至织金段全面完工，白云至修文城市干道建成通车，客运东站新站投入使用。启动中心城区畅通工程二期，推进1.5环建设和城市快速公交（BRT）项目前期工作，轨道交通1号线全面开工，2号线前期工作有序推进。龙洞堡机场1号航站楼改造工程基本完成，新增、加密国际航线6条，旅客吞吐量突破1200万人次。“5个100工程”完成投资1166.21亿元。遵义、安顺、贵安新区与贵阳实现通信同城化。

　　一年来，我们取得了一批实实在在的生态文明建设成果。“蓝天”“碧水”“绿地”三大保护计划深入实施，森林覆盖率45%，空气质量优良率86%，集中式饮用水源水质稳定达标。开展环境空气质量专项治理，完成中心城区天然气置换，淘汰黄标车1.43万辆，淘汰落后产能82.4万吨，二氧化碳减排43.78万吨。完成饮用水源地一级保护区核心区居民搬迁，全面实施南明河水环境综合整治二期工程，建成污水处理厂4座、提标改造7座。获批全国水生态文明建设试点城市。建成孔学堂二期，改造提升花溪十里河滩国家城市湿地公园、黔灵山公园，建成区绿化覆盖率达44%。基本完成饮用水源、城市通风走廊、森林资源保护等红线划定工作，全省首单中国自愿减排（CCER）项目成功签约，实现碳交易零突破。开展“六个一律”环保“利剑行动”、森林保护“六个严禁”专项执法行动，查处生态环保案件666起。

　　一年来，我们办成了一批惠及民生的实事。启动解决“民生十困”10项行动计划，办好“十件实事”。民生支出占公共财政支出的62%。基本解决50余个城市重点缺水片区用水问题。城镇新增就业21.12万人，农村劳动力转移就业3.18万人。新增幼儿园及中小学学位4.47万个，与北京等地20所著名优质学校合作办学，贵州（清镇）职教城入驻院校19所。组建贵阳市医院管理（集团）有限公司。建立全科医生团队52支，改扩建10个乡镇卫生院、50个村卫生室，完成8个社区卫生服务中心标准化建设。人口计生工作继续实现“双降”目标，在全省综合考核评比中连续21年保持一等奖第一名。建成一批养老院、敬老所，新增床位4158张。实施全民健身工程460个。城乡居民最低生活保障标准均提高11%以上。减少贫困人口3.15万，180个贫困村“减贫摘帽”。基本建成城镇保障房4.65万套。完成棚户区改造项目43个、惠及5.6万人。启动老旧居民小区改造升级“三年千院”行动计划，实施公益事业项目1457个。建成农产品二级批发市场2个、中心城区生鲜直销超市8个。居民消费价格指数102.7，物价保持总体平稳。“四在农家·美丽乡村”基础设施建设六项行动计划完成投资25.63亿元，建成53个“提高型”示范点，形成3条示范带。改造提升村级综合楼136个。

　　过去的一年，我们妥善处置“2·27”公交车放火案等突发事件，科学应对“7·16”特大洪灾。深入开展“两严一降”，打响禁毒人民战争，完善立体化社会治安防控体系，全市刑事发案数下降21.1%，群众安全感提升2.96个百分点、达97.16%。扎实抓好信访维稳“一号工程”，排查化解信访积案1762件，化解率97.1%。成功举办“创新社会治理、加强社会建设”2014（贵阳）年会。深化安全生产大检查和专项整治，事故起数和死亡人数继续实现“双降”。全面完成第三次全国经济普查。国防、人防、国家安全、食品药品安全、审计、司法行政、民族宗教、移民、地方志、档案、侨务、对台、气象、老龄、工青妇、关心下一代、慈善、残疾人、红十字、爱国卫生等各项事业全面发展。

　　过去的一年，我们扎实开展党的群众路线教育实践活动，切实纠正“四风”，严肃查处违纪违法案件，给予党纪政纪处分454人。严格执行中央、省委、市委规定，厉行节约，压缩行政经费8%用于教育支出。办理人大代表建议197件、政协提案360件，办结率和满意率均为100%。提请市人大常委会审议地方性法规草案4件，制定政府规章12件，依法办理各类行政复议186件。

　　过去的一年，面对经济发展新常态，我们坚决维护中央和省委权威，坚决服从市委领导，切实做到“市委有号召，政府有行动；市委有部署，政府有落实”，按照“统起来、严起来”的要求，切实转变职能、理顺机制，强化统筹、精细调度，钉钉子、拧螺丝，通过一桩桩事情公平公正的解决，一项项工作务实有效的推进，一件件大事难事积极稳妥的处置，做好市委正确决策的“证明题”，努力创造经得起实践和人民检验的实绩！

　　各位代表，过去一年的成绩来之不易，这是省委、省政府和市委正确领导的结果，是省级单位和兄弟市（州）大力支持的结果，是市人大、市政协监督支持的结果，是全市干部群众团结奋斗的结果。在此，我代表市人民政府，向全市各族人民，向各位人大代表和政协委员，向各民主党派、工商联、无党派人士、各人民团体和各界人士，向驻筑人民解放军、武警官兵和人民警察，向中央及省在筑单位，以及所有关心、支持贵阳建设发展的各位老领导、老同志和各界朋友，表示衷心的感谢!

　　成绩已成过去，未来更具挑战。从经济运行看，下行压力持续加大，特别是对稳定增长具有关键作用的投资增速放缓，个别主要经济指标未实现预期目标，增长动力转换青黄不接；从市场主体看，部分企业生产经营困难，“融资难、融资贵”问题突出；从风险防控看，政府偿债压力较大，非法集资等潜在隐患开始显现；从民生改善看，城乡二元结构、城市内部二元结构矛盾还未得到有效化解，人口资源环境矛盾依然存在；从政府自身看，有的地方和部门担当不够，作风不实、效率不高、落实不力的问题还一定程度存在，一些决定事项未按期完成。对这些问题，我们将坚持用发展的办法、改革的举措和扎实的作风切实加以解决。

　　二、2015年工作安排

　　今年是全面完成“十二五”规划的收官之年、全面深化改革的关键之年、全面推进依法治国的开局之年，也是我市在全省率先实现全面小康的决胜之年。当前，全市经济社会发展进入了坚守两条底线、推进开放创新、加速“四化”同步、实现跨越增长的新常态，既迎来大数据时代、高铁时代、区域合作时代等新机遇，又面临经济发展、开放竞争、技术革新、城市治理等新挑战，贵阳必须扮演好变革者、带动者、示范者、开创者的新角色，在新起点上全面打造贵阳发展升级版。在经济发展新常态的重要战略机遇期，我们走的不是逐步工业化的老路，也不是先污染后治理的弯路，而是创新驱动、转型升级的新路，既有量的显著扩张，更有质的巨大飞跃，我们完全有条件、有能力、有信心、有底气，处理好经济发展和生态保护的关系，让习近平总书记对贵州守住两条底线的指示要求在贵阳落地生根，指引我们在“双赢之路”上后发赶超！

　　今年政府工作的总体要求是：全面贯彻党的十八大和十八届三中、四中全会精神，以邓小平理论、“三个代表”重要思想、科学发展观为指导，深入贯彻习近平总书记系列重要讲话精神，按照中央、省委、市委的各项决策部署，牢牢守住发展和生态两条底线，把握新机遇、引领新常态，紧扣主基调、实施主战略，加快改革推动、开放带动、创新驱动，集中力量实施“六大工程”，着力打造开放贵阳、创新贵阳、生态贵阳、法治贵阳、人文贵阳、和合贵阳升级版，在全省率先实现全面小康，以方向更准、速度更快的“火车头”和质量更高、效益更好的“发动机”带动全省经济社会发展，加快建设全国生态文明示范城市，朝着率先基本实现现代化的目标大踏步前进。

　　全市经济社会发展的主要预期目标是：生产总值增长13.5%左右，500万元口径固定资产投资增长20%，2000万元口径规模以上工业增加值增长13%，社会消费品零售总额增长13%，进出口总额增长18%，公共财政预算收入增长14%，城镇、农村常住居民人均可支配收入分别增长10%和13%，居民消费价格涨幅控制在省调控目标范围内，万元生产总值综合能耗、主要污染物排放总量控制在省下达目标范围内，空气质量优良率保持在80%以上。

　　今年13.5%左右的经济预期增速，比全国、全省分别高6.5个、3.5个百分点。在宏观经济下行压力加大的情况下，贵阳和其他地区一样会受到较大影响，特别是我们支撑发展的产业基础、要素条件、政策环境正在发生深刻变化，要实现13.5%左右的目标，难度很大，压力不小，需要我们努力拼搏，需要我们共克时艰，抓住用好新机遇，培育增长点、管控风险点，用抓铁有痕、踏石留印的行动，狠抓各项工作的落实。

　　面对新形势和新任务，我们要在市委的坚强领导下，坚持加快发展不犹豫、开放创新不动摇、为民务实不放松，努力创造贵阳发展升级的八个“加速度”。

　　（一）创造投资消费加速度，努力保持经济稳定增长。我们要从培育需求方面施策，从增加供给方面使力，充分发挥投资的关键作用和消费的基础作用，积极培育出口新增长点，保持好当前来之不易的发展势头。

　　一是千方百计扩大有效投资。注重调结构、补短板、惠民生，抢抓国家实施生态环保、信息网络、铁路交通等七类重大工程建设机遇，积极谋划实施一批支撑产业发展的大项目、好项目。采取投资补助、基金注资、担保补贴等方式，鼓励民间资本参与重大工程建设，力争民间投资达2200亿元。集中开展重大招商项目建设攻坚战，全力抓好手续审批、房屋征收、项目融资等保障服务，开工建设吉利甲醇汽车等62个重点项目，建成惠普贵州国际金贸云基地等14个项目。确保199个省重大工程和重点项目完成投资1200亿元，“5个100工程”完成投资1000亿元，全社会固定资产投资突破4000亿元。

　　二是千方百计培育消费热点。深入实施城乡居民收入倍增计划，让群众能消费、愿消费、敢消费。充分把握个性化、多样化的消费趋势，办好酒博会、茶博会、农产品交易会等重大展会，瞄准养老、健康、家政、信息、旅游、住房、教育、文化、体育等潜力大、前景好的领域，积极培育新的消费增长点，运用电商等新兴商业模式，刺激消费，扩大内需，力争社会消费品零售总额突破1000亿元。

　　三是千方百计挖掘出口潜力。充分发挥贵阳综合保税区功能，加快跨境电子商务发展平台和电子口岸建设，申报创建国家服务外包示范城市、跨境电子商务试点城市。实施“外贸、外资、外经企业培育工程”，培育5家以上进出口总额超过1亿美元的外贸企业、1家进出口总额超过1000万美元的外资企业、2家对外经济合作超过1亿美元的外经企业，提高贵阳产品外销率，力争全年外贸进出口总额突破100亿美元，其中服务贸易20亿美元。

　　（二）创造创新驱动加速度，努力推动产业转型升级。我们要把创新驱动放在经济发展全局的核心位置，以大数据为引领，深入实施“百千万工程”和“十百千计划”，加速推进产业转型发展。

　　一是推动现代工业集群发展。加快创建国家级大数据产业发展集聚区。坚持互联互通、开放共享，以城市公共大数据平台建设和智慧贵阳云应用示范为抓手，以政府数据开放和市场数据交易为内生动力，以具有比较优势的数据中心和呼叫中心产业为切入点，努力构建全产业链的大数据产业生态环境。启动实施“3366工程”，联手富士康、阿里巴巴、世纪互联等业内知名企业，加快谋划实施大数据基础平台、高端制造等重点工程项目，建成全域公共免费WiFi城市项目一期工程，打造块上集聚的城市大数据公共平台，组建大数据交易所，推进数据安全保障体系建设和大数据安全制度创新。着力打造“全球呼叫·贵阳服务”的服务外包品牌，新增呼叫中心5万坐席，力争形成10万坐席规模。开展智慧贵阳云应用示范，统筹推进大数据在多个领域的示范应用，积极创建“国家智慧城市”。深入实施“强基、筑云、智端、掘金”四大工程，推动大数据产业与其他产业的深度融合和创新运用，引领三次产业加快转型升级。力争大数据产业规模总量达1000亿元。加快打造医药健康产业集群。坚持“四轮驱动、四个结合”，大力发展化学药、中药深加工、生物制药、医学工程，全力建设贵阳医药产业园，扶持壮大益佰、同济堂、景峰等一批重点企业，加快国药集团骨科中药生产基地、悦康医药产业园及博奥生物、神奇医养园等项目建设，推动修正药业、中卫莱康、北科生物等龙头企业落户贵阳，实现医药健康产业产值280亿元。加快打造特色产业集群。以新能源汽车、服务器制造等为重点，大力发展高端装备制造业。深入实施“四个一体化”，建成投产开磷煤电磷、中铝煤电铝、贵钢煤电钢等项目。支持开阳新型建筑材料产业园建设，全市新型建筑建材业产值增长30%。加快白云国家“城市矿产”示范基地建设。继续抓好食品、卷烟、茶叶等特色产业，提升老干妈、贵烟等品牌价值，“六大特色产业”增加值超过550亿元。建成投产康师傅饮品、海跃模具等100个千万元以上的工业投资项目，新增上规工业企业50户，规模以上工业增加值突破700亿元。

　　二是推动现代服务业集聚发展。以打造西部科技金融创新城市和互联网金融创新城市为载体，抓好全国移动金融试点工作，推进贵阳互联网金融产业园建设，引导各类互联网金融业态集聚发展。加快贵阳国际金融中心建设，新引进金融机构2家、设立科技银行2家，确保金融业增加值增长18%。以打造全省旅游集散中心为目标，大力实施景区改造升级、旅游配套服务提升、旅游市场拓展等工程，建成一批旅游集散服务中心、特色旅游文化街区和精品酒店，全面推进花溪文化旅游创新区建设，力争青岩古镇创建为国家5A级旅游景区，旅游总收入增长19%。以打造现代服务业集聚区为重点，持续推进中心城区批发业态转移，启动建设新市西商业街，加快建设西南国际商贸服务业集聚区，规划建设贵阳火车北站商务区，优化提升中心城区商务区，大力发展总部经济。开工建设“马上到”国际物流园二期，建成投运传化智能公路港、京东电商产业园一期。全年电商交易额突破500亿元，物流业增加值达170亿元。

　　三是推动都市现代农业加快发展。以市场需求为导航灯，以资源禀赋为定位器，用抓工业的方式抓农业，促进一二三次产业融合互动。加强现代农业高效示范园区建设，支持贵州开阳台湾产业园加快发展，办好首届农业嘉年华活动。支持修文猕猴桃产业做优做强。大力发展农业领域电子商务，不断创新农产品营销模式。大力发展新型农业经营主体，力争引进伊利、正大等知名龙头企业，培育扶持农产品加工企业30家、家庭农场100个、村级集体经济组织150个，基本消灭集体经济空壳村。农产品加工业产值增长20%以上，现代农业增加值比重30%以上。

　　四是以完善创新体系为抓手推进创新要素集聚。围绕申报创建国家自主创新示范区，推动中关村6项先行先试政策落户贵阳，深化全国知识产权示范城市和全国质量强市示范城市建设，实施“六个科技行动”，构筑大众创新创业生态体系。建设戴尔贵阳实验室和国际华人科技工商协会贵阳国际创新中心、贵阳大数据创新中心、大数据创新创业孵化器，组织开展贵州大数据产业创新创业大赛。用好“一站一台”“一部一院”、贵阳高校科研院所科技创新联盟等平台，强化企业创新主体地位，大力推进技术攻关、成果转化。加快贵州科学城、中国海外高端人才创新创业（贵阳）基地建设，创新人才引进机制，推动形成招商引人才、人才促产业、产业再聚才的发展模式。全市高新技术企业超过200家，全社会研发支出占生产总值比重达1.5%以上。

　　（三）创造破解民生难题加速度，努力提高群众幸福指数。让全市人民过上好日子，是我们的根本目标。我们将以更大的投入、更有力的举措，察民情、知民意、解民忧、纾民困，全力破解“民生十困”、办好“十件实事”，让群众生活得更加幸福。

　　一是着力解决“用水难”问题。启动花溪碧云窝水厂扩容工程，完成阿哈水库至中曹水源工程、鱼洞峡水厂管网与市政供水管网连通工程，推进一户一表改造工作，不断完善城市供水保障体系。二是着力解决“就业难收入低”问题。建成高技能人才培训基地9个，新建市级以上示范创业孵化园4个，大力实施“3个20万”延伸行动计划等创业鼓励政策。城镇新增就业22万人，农村劳动力转移就业2.8万人，新增创业实体3.7万户，城镇登记失业率控制在4%以内。继续推进社会保障提标扩面，城乡最低生活保障标准提高10%以上，基本社会保险覆盖率90%以上。三是着力解决“上学难上好学难”问题。坚持“教育立市”不动摇，奋力完成教育“9+3”计划，落实“1+6”教育配套政策，确保义务教育巩固率达95%，高中阶段毛入学率达90%，实现县域义务教育初步均衡，力争8个区（市、县）基本普及十五年教育。巩固扩大京筑合作办学成果，加大开放合作办学力度。继续实施学前教育三年提升行动，全市87%的乡（镇、社区）拥有公办或普惠性幼儿园。加快贵州（清镇）职教城、省级示范高职院校建设。推进农村义务教育阶段学生营养餐计划。继续压缩8%行政经费用于教育。四是着力解决“出行难”问题。成立城市交通道路应急处置中心，加强智能交通管理系统建设，对200多个周期性、常发性交通堵点采取专门应急处置和防范措施。搬迁金阳临时客运站。加强出租车行业管理，严厉整治非法营运。五是着力解决“看病难”问题。积极推进城市公立医院改革，全面实施县级公立医院综合改革，鼓励和有序引导社会办医。扎实抓好全科医生执业方式和服务模式改革试点，每万人、每个乡镇卫生院至少有1名全科医生。加强基层医疗卫生机构标准化建设，改扩建标准化中心乡镇卫生院10个，实施6个社区卫生服务中心标准化建设。建立市级突发公共卫生事件应急指挥决策平台。加快公共卫生救治中心建设。建立城乡居民重特大疾病医疗救助制度，切实发挥助困兜底作用。把控制人口增长、提高人口素质作为守住两条底线的重要基础和内在要求，全面加强人口计生工作。六是着力解决“养老难”问题。抓好全国养老服务业综合改革试点工作。完善政府供养制度，保障孤老优抚对象、城市“三无”老人、农村“五保”老人基本生活。基本建成以居家为基础、社区为依托、机构为支撑的覆盖城乡的养老服务体系，每千名老年人拥有养老床位30张以上。七是着力解决“治安差”问题。围绕建设“安全城市”，持续深入开展“两严一降”和“禁毒人民战争”，筑牢社会治安综合治理网底，进一步降低刑事发案数，提高群众安全感。八是着力解决“住房难”问题。加快推进保障性住房建设，建成公共租赁住房1.6万套。探索建设“新微公共租赁住房”，解决新就业大中专学生和产业园区技术工人阶段性住房困难。九是着力破解“买菜难买菜贵”问题。完善农副产品“保供稳价”体系，划定城市保供菜地红线，规划建设10万亩蔬菜应急储备基地、1万亩标准化蔬菜示范基地，推进3个产地型农产品交易市场、3个中心城区公益性菜市场建设。开展“肉霸”“菜霸”专项打击行动，进一步整顿和规范农副产品市场经营秩序。十是着力解决“活动空间少”问题。新建一批小广场、小游园，改造一批社区院落，提升一批城市公园，配套完善文化体育设施，新增城市活动空间40万平方米。

　　（四）创造深化改革加速度，努力激发经济发展内生动力。我们要坚持问题导向，聚焦经济社会发展的重点领域，聚焦群众高度关注的热点难点问题，蹄疾步稳，统筹推进，确保“改有所进、改有所成”。

　　一是深化行政管理体制改革。勇于自我革命，积极打造大数据时代政府职能转变“升级版”，建设服务型、责任型政府。以大数据等方式完善“权力清单”“负面清单”“责任清单”制度，推进简政放权，抓好“接放管”，打通“中梗阻”和“最后一公里”，让数据多跑路、群众少跑腿。以大数据等方式推动政府治理能力现代化，加快聚集社会治安、社区服务、教育卫生等民生数据资源，构建科学精细的社会公共服务系统。

　　二是深化财税体制改革。全面落实国家财税体制改革总体方案，严格执行新《预算法》，构建跨年度预算平衡机制，实行全面规范、公开透明的预决算管理制度，公开所有使用财政资金的部门预决算。全面推进“营改增”，健全地方税收征管体系，清理规范税收等优惠政策。进一步完善市区财政管理体制。

　　三是深化投融资体制改革。积极推广政府与社会资本合作（PPP）等多种投融资模式，大力发展股权和创业投资基金，鼓励民间资本发起设立产业投资基金，积极支持企业上市融资。进一步规范政府融资工作，完善“借、用、还”一体化体系，实行“银行、平台公司、政府”三方监管，探索建立政府性融资纳入预算资金管理制度。

　　四是深化城市开发模式改革。强化用地统筹，进一步坚持、完善土地出让和供给“六统一”制度，严格执行“净地”出让，通过“统一规划、统一收储、统一出让、统一交易平台、统一出让金收缴、统一监管”，把握土地供应规模和节奏，提高城镇建设用地利用效率，推进城市开发建设可持续发展。

　　五是深化国有企业改革。以市场化和股权多元化为方向，鼓励社会资本参与国有企业改革，大力发展混合所有制经济，完成市属平台公司实体化转型和80%以上市属国有实体企业混合所有制改革。完善以管资本为主的国有资产监管体制，做实市属国有企业产业发展基金，支持国有企业改组为国有资本投资公司。探索建立市属国有企业职业经理人制度。

　　六是深化农村产权制度改革。全面完成农村土地承包经营权确权登记颁证，继续推进果树、茶树经营权、集体建设用地使用权、宅基地、农村房屋确权登记颁证工作。成立市级农村综合产权交易中心，在“一市三县”成立服务中心。开展流转经营权颁证试点，推动农村土地“三权分置”、有序流转。开展农村土地承包经营权、宅基地、农村房屋产权抵押担保融资试点。

　　全力抓好上海自贸试验区可复制改革试点经验的落地工作，对投资管理领域、贸易便利化领域、金融领域、服务业开放领域和事中事后监管措施5个方面、29项改革事项，进行逐项研究并分解责任，确保改革试点经验在贵阳落地见效。完成全市机关公务用车制度改革，实施机关事业单位养老保险制度改革，推进司法体制改革试点，启动居住证制度改革试点，继续推进其他领域改革。

　　（五）创造开放带动加速度，努力提高开放型经济发展水平。我们要进一步解放思想，抢抓机遇，积极加强对外交流合作，推动区域融合发展，以开放聚集要素，以开放促进转型。

　　一是把平台打造作为扩大开放的主要抓手。坚持以中关村贵阳科技园为统揽，着力形成高新区、经开区、综保区、双龙临空经济区“四轮驱动”开放格局，带动全市产业园区加快发展。启动贵阳综保区二期、《中瑞自由贸易协定》贵阳示范园建设，用好用足国家赋予的特殊政策，强化综保区保税加工、物流、贸易、金融等核心功能。加快双龙临空经济区建设，确保基础设施精彩亮相，产业集聚初见成效，积极申报创建国家级临空经济区，完成贵阳航空口岸改造。加快高新区、经开区建设，展示中关村贵阳科技园核心区新形象，推进白云区、高新区、综保区、修文县同城化发展，深化经开区与花溪区融合发展。全力办好2015贵阳国际大数据产业博览会暨全球大数据时代贵阳峰会，率先发布大数据交易相关标准。当好“东道主”，举全市之力，细致、精致、极致办好生态文明贵阳国际论坛，用好这个制高点，抓住论坛进入国家“一带一路”发展规划的机遇，使其在全省开放带动、创新驱动中发挥更大作用。建设好、管理好、发展好孔学堂，充分发挥海峡两岸交流基地作用，让孔学堂与生态文明贵阳国际论坛并驾齐驱，打造两个对外开放合作的制高点。办好酒博会、中国—东盟教育交流周等活动，用好用活对外交流合作的“新视窗”。

　　二是把区域合作作为扩大开放的主攻方向。深化京筑创新驱动、区域合作，突出资源引进和承接产业转移，巩固扩大“总部在北京、基地在贵阳”合作成果。抢抓贵广高铁开通重大机遇，主动融入泛珠三角区域发展，努力把贵阳打造成为贵广高铁经济带重要增长极。加快建设开阳港和“北海贵阳港”，主动对接蓉欧、渝新欧国际铁路，积极参与“一带一路”、长江经济带建设，借港入海、借道出山。积极推进黔中经济区同城化发展。

　　三是把招商引资作为扩大开放的首要任务。精心办好2015（贵阳）布鲁塞尔国际烈性酒大赛、2015年中国材料大会等重大活动，积极组织和参与贵州·北京大健康医药产业推介会等活动，突出专题招商、行业招商、定向招商、领导带头招商，实际利用外资增长20%，力争突破10亿美元，省外实际到位资金增长20%，总量突破2700亿元。

　　（六）创造新型城镇化加速度，努力推进城乡一体化发展。我们要加快山地特色新型城镇化步伐，坚持建管并举、城乡并重，兼顾社会效益、生态效益、经济效益，不断提高城乡一体化发展水平，打造宜居宜业宜游的“爽爽贵阳”。

　　一是以组团式发展为方向，加快“疏老城、建新城”。坚持“组团式规划、功能性布局、协同化发展”，开展城市开发边界划定试点工作。大力推进“六规合一”，完善树形规划层级结构系统，以人为本、优化布局，着力提高城市规划和建筑风貌形象设计水平，统筹规划与贵安新区相邻的花溪、清镇城市建筑风格，发展有历史记忆、地域特色的美丽城镇，不断提升城镇品质和内涵，努力打造“山水风光、精致品格、西部风情”的特色城市。妥善处理城市发展、民生改善和资源环境承载能力矛盾，有效防止“大城市病”。按照产城互动、教城互动的思路，合理规划建设一批城市组团，带动老城改造。坚持“多拆少建”“只拆不建”，实施棚户区改造攻坚行动，加快旧城改造步伐，统筹推进新区建设，让老百姓既能安居、又能乐业。抓好三马片区等39个棚户区改造项目，推进商专茶店校区、贵大花果园校区和沙冲路校区搬迁工作，进一步疏解老城区市场、教育、卫生和行政功能，努力改善老城区居住条件和环境面貌。办好2015年全国城市规划年会。

　　二是以中心城区畅通工程二期为重点，构建立体交通体系。加快推进朝阳洞路、南垭路、黔春大道建设，力争早日建成1.5环。积极创建“公交都市”，抓好城市快速公交（BRT）、公交停保场站和老城区公交专用道建设。加快轨道交通1号线建设，全面启动2号线（一期）、S2号线。继续加密城市路网，建成沙文至扎佐、金清联络线、北京东路延伸段一期，适时启动北京东路延伸段二期，推进宾阳大道延伸段、210国道改造、观潭大道等重点工程，促进城市交通由“中心聚集”向“环网分担”转变。加快市域快铁和省际高铁建设，尽快开通运营市域快铁贵阳至开阳客运专线、白云至龙里段建成通车，沪昆客专贵阳至长沙段贯通。争取贵阳至南宁客专开工建设，贵阳至襄阳客专铁路项目纳入国家“十三五”规划。力争启动龙洞堡机场三期工程，继续推进贵阳至瓮安、贵阳至黔西等7条高速公路建设。

　　三是以改善环境秩序为抓手，提高城市管理水平。巩固“创文”“创卫”成果，实施城市管理巩固提升三年行动计划，广泛开展绿化、美化、亮化、净化工作，强化“条专块统”，建立环境卫生秩序长效管理机制。切实加大“脏乱差”整治力度，改造提升一批城市破旧道路，加强垃圾处理设施建设，提高城市垃圾无害化、减量化、资源化处理水平。强化城市综合执法。建立完善控违拆违长效机制，保持打击违法建筑高压态势，确保违法建筑“零增长”。

　　四是以破解二元结构矛盾为目标，促进城乡一体化发展。持续壮大县域经济，力争县域经济总量占全市比重提高0.5个百分点，确保“一市三县”通过同步小康创建达标认定。加快红岩水库、那卡河水库等骨干水源工程建设，开工建设息烽高洞、开阳杉木林等一批中小型水库。加快花溪孟关等4个风情小镇建设，新启动建设5个。推进城市基础设施和公共服务向农村延伸，坚持产城景融合，强化村镇规划，探索建立“1+N”镇村联动发展模式，启动30个示范点建设。深入实施“田园保护”计划，集中改造一批村容村貌。加大“四在农家·美丽乡村”建设力度，建成40个“提高型”示范点。坚持“三位一体”精准扶贫，确保1.44万贫困人口脱贫、150个贫困村“减贫摘帽”，扶贫生态移民搬迁3000人。

　　（七）创造生态建设加速度，努力建设生态文明美好家园。生态是贵阳最大的优势，但基础又十分脆弱，我们要坚持生态优先，巩固“创模”成果，深入实施“蓝天”“碧水”“绿地”三大保护计划，努力实现百姓富、生态美的有机统一。

　　一是大力建设生态环境。按照建设公园城市的部署，抓好“千园”布局规划，建设东山等山体公园，提升阿哈湖等湿地公园，改造长坡岭等森林公园，加快构建“五位一体”城市公园体系。大力提高城市绿化水平，实施示范工程6个，绿化改造社区院落200个，完成环城绿化1.8万亩，新增城市绿地100万平方米。实施退耕还林、天然林保护、石漠化治理等重点工程，完成营造林16万亩，治理水土流失20平方公里，湿地保有量24万亩，森林覆盖率提高到45.5%。

　　二是大力防治环境污染。深入实施环境空气质量攻坚计划，扎实推进无煤城市建设，积极开展绿色低碳循环交通运输体系区域性试点工作。加快国家水生态文明试点城市建设，依法推进饮用水源保护区居民生态搬迁，完成南明河综合整治二期工程，新建三桥、小关等10座小型污水处理厂和10个雨水利用项目，实施2个污水再生利用试点工程。加强生态功能区管控，加快破碎山体生态修复。

　　三是大力加强制度建设。制定实施城市环境总体规划，严守环境承载上线、生态保护红线、环境风险底线。严格执行新修订的《中华人民共和国环境保护法》《贵阳市建设生态文明城市条例》，深入开展“六个一律”环保“利剑行动”、森林保护“六个严禁”专项执法行动，从严从重打击破坏生态环境违法行为。进一步加强环保执法力量，完善乡镇生态环保机构，探索组建生态建设投资公司。率先在全省建立领导干部政绩考核与水、空气、土壤3个环境质量指标挂钩制度，强化对区（市、县）生态环境保护的责任落实和考核监督，把做好生态保护作为各级政府和基层组织最大的“政绩”，引导全市上下在打造贵阳发展升级版的进程中，更加自觉地建设生态、保护环境，确保环境质量稳中有升，为全省生态文明建设探路子、作示范。

　　（八）创造社会事业发展加速度，努力促进社会和谐稳定。我们要大力发展社会事业，推进经济社会协调发展，促进社会公正、增进人民福祉。

　　一是加快发展文体事业。系统构建以孔学堂、阳明洞、阳明祠三足“鼎”筑的贵阳“精神大厦”，更加自觉主动地推进孔学堂事业发展，进一步完善孔学堂教化、传播和研修功能，坚持创造性转化和创新性发展，把孔学堂打造成为传承弘扬中华优秀传统文化，培育和践行社会主义核心价值观的重要教育基地，在凝聚传递贵阳发展的正能量中发挥更大作用。建成阳明文化园核心区，实施甲秀楼、文昌阁、阳明祠等24个城市历史文化街区建设工程。广泛开展诚信创建活动，大力弘扬“知行合一，协力争先”的贵阳精神，构建精神高地、打造文化品牌。加快完善公共文化设施，实现图书馆和文化馆区（市、县）全覆盖、综合文化站乡（镇）全覆盖，成功创建国家公共文化服务体系示范区。举办“华文媒体论坛”，深入开展“三下乡”、电影“五进”、群众周末大舞台等群众性文化活动，满足市民多样化文化需求。大力推进文体融合发展，建设奥体中心二期等一批项目，办好2015贵阳国际田联世界越野锦标赛、贵州省第九届运动会、贵阳国际马拉松等重大赛事。

　　二是推进社会治理创新。巩固提升城市基层管理改革“贵阳经验”，依法完善社区服务机构职责，理顺关系，做强社区、做实居委会、做精网格，健全基层社会治理体系。切实管控好风险点，严防政府债务、金融、房地产等引发风险，以及因经济问题引发的各种社会矛盾。抓好信访维稳“一号工程”，推进维护稳定工作指挥调度中心建设，落实安全生产责任制，强化食品药品安全监管，加强公共安全和应急管理，守土有责、守土有效，切实维护社会安全和谐稳定。

　　三是推进各项社会事业全面发展。继续推进民族宗教、外事侨务、对台、仲裁、地方志、档案、气象、老龄、残疾人、国家安全等事业发展，支持国防建设和法院、检察院工作，支持工青妇、关工委、红十字会、计生协会等组织做好工作。同时，我们要在深入贯彻落实国发2号文件的基础上，精心编制“十三五”规划，为在全省率先实现基本现代化谋好篇、布好局。

　　三、运用法治思维和法治方式推进政府自身建设

　　我们要巩固和拓展党的群众路线教育实践活动成果，继续找问题、找差距、找目标，着力抓统筹、抓落实、抓提升，紧紧围绕全面推进依法治市，将法治思维和法治方式贯穿于政府工作各个方面，坚持对上负责与对下负责相统一，努力建设组织放心信任、人民满意信赖的政府。

　　一是始终把依法行政作为基本遵循，加快建设法治政府。忠实履行宪法和法律赋予的职责，按照职能科学、权责法定、执法严明、公开公正、守法诚信的要求，把政府工作全面纳入法治轨道。进一步完善政府重大事项科学决策机制，把公众参与、专家论证、风险评估、合法性审查、集体讨论决定贯穿于重大行政决策全过程，坚持政府法律顾问制度、决策跟踪反馈和纠错问责制度。严格规范文明公正执法，大力推进综合执法。坚持对人大负责、受人大监督，自觉接受人大及其常委会的法律和工作监督，依法向人大报告环境保护等工作完成情况。自觉接受政协民主监督、群众社会监督和新闻舆论监督。

　　二是始终把作风建设作为永恒主题，加快建设服务政府。坚定理想信念，严守党的政治纪律和政治规矩，切实做到心中有党、心中有民、心中有责、心中有戒，建设一支忠诚、干净、担当的公务员队伍。深入落实中央八项规定、省委十项规定和市委二十九条实施细则，认真践行“三严三实”，按照“抓常、抓细、抓长”的要求，推进作风建设常态化，驰而不息纠正“四风”。积极面对新形势下的新挑战、新任务，遇到矛盾不绕道，遇到问题不回避，坚持战略定力、省会标准、脚踏实地、群众路线，提升执行力，做好“证明题”，以钉钉子、拧螺丝的精神，紧盯目标抓落实、紧盯政策抓落实、紧盯项目抓落实、紧盯企业抓落实，真抓实干，勇于担当，拿数据说话，靠作风吃饭，用我们的辛苦指数换取老百姓的幸福指数。

　　三是始终把廉洁从政作为履职底线，加快建设廉洁政府。严格落实党风廉政建设“两个责任”，坚持“一岗双责、守土有责”，确保经济社会发展和党风廉政建设各项任务落实。从严治政，严管队伍，始终坚持对腐败“零容忍”，加大对群众身边不正之风和腐败问题查处力度，发现一起查处一起，做到有腐必反、有贪必肃。全面推进惩治和预防腐败体系建设，用大数据编织制约权力的“笼子”。厉行勤俭节约，反对铺张浪费，加强“三公”经费管理，节约政府开支，降低行政成本。严肃财经纪律，加强审计监督，确保公共资金使用安全，促进干部清正、政府清廉、政治清明。

　　各位代表！创新成就未来，实干铸就辉煌，让我们紧密团结在以习近平同志为总书记的党中央周围，在省委、省政府和市委的坚强领导下，团结依靠全市各族人民，坚定信心，排难而进，抢抓机遇，奋发有为，全面打造贵阳发展升级版，为率先在全省基本实现现代化而努力奋斗。
